# A Computational Histology Artificial Intelligence Prognostic Biomarker in Non-Small Cell Lung Cancer Using the Cancer Genome Atlas

**DOI:** 10.3390/cancers18182916

**Published:** 2026-09-09

**Authors:** Benjamin A. Bleiberg, Masaoki Ito, Melina Marmarelis, Viswesh Krishna, Vivek Nimgaonkar, Haochen Zhang, Trevor J. Royce, Yasuhiro Tsutani, David Kozono, Charu Aggarwal

**Affiliations:** 1 Division of Hematology-Oncology, Department of Medicine, University of Pennsylvania, Philadelphia, PA 19104, USA; benjamin.bleiberg@pennmedicine.upenn.edu (B.A.B.); charu.aggarwal@pennmedicine.upenn.edu (C.A.); 2 Abramson Cancer Center, University of Pennsylvania, Philadelphia, PA 19104, USA; 3 Division of Thoracic Surgery, Department of Surgery, Kindai University, Osaka 577-8502, Japan; 230400@med.kindai.ac.jp; 4 Penn Center for Cancer Care Innovation, University of Pennsylvania, Philadelphia, PA 19104, USA; melina.marmarelis@pennmedicine.upenn.edu; 5 Valar Labs, Palo Alto, CA 94306, USA; viswesh@valarlabs.com (V.K.); vnimgao2@jh.edu (V.N.); haochen@valarlabs.com (H.Z.); 6 Sidney Kimmel Comprehensive Cancer Center, Johns Hopkins University, Baltimore, MD 21287, USA; 7 Department of Radiation Oncology, University of North Carolina at Chapel Hill School of Medicine, Chapel Hill, NC 27514, USA; 8 Division of Thoracic Surgery, Kindai University, Osaka 589-0014, Japan; tsutani@med.kindai.ac.jp; 9 Dana Farber Cancer Institute, Boston, MA 02215, USA; dkozono@bwh.harvard.edu

**Keywords:** non-small cell lung cancer, digital pathology, artificial intelligence

## Abstract

**Background/Objectives**: Outcomes in non–small cell lung cancer (NSCLC) remain heterogeneous, even within stage and molecular subtypes. We evaluated whether a Computational Histology Artificial Intelligence (CHAI) biomarker, derived solely from the diagnostic hematoxylin-and-eosin (H&E) whole-slide image (WSI), provides prognostic information independent of established clinicopathologic factors. **Methods**: Using The Cancer Genome Atlas (TCGA) lung adenocarcinoma (LUAD) and lung squamous cell carcinoma (LUSC) projects, 914 patients with an evaluable diagnostic digitized whole-slide images (WSI) and outcomes data were stratified by stage and histology and randomly split into a development set (30%, N = 270) and a held-out validation set (70%, N = 644). A CHAI histologic signature was developed and locked on the development set and applied without modification to the validation set. In development, image features were extracted from H&E-stained WSIs and used to train a Cox proportional hazards model to output a CHAI prognostic biomarker score and then dichotomized into high (CHAI positive (+))- and low (CHAI negative (−))-risk groups. The primary endpoint was overall survival (OS), and the secondary endpoint was progression-free interval (PFI). Associations were assessed by Kaplan–Meier and Cox proportional-hazards models and adjusted for traditional clinicopathologic and genomic variables. **Results**: A total of 644 patients were included in the validation cohort. Median age was 68 (IQR: 60–74), 388 (60%) were male, 317 (49%) had LUAD, 327 (51%) had LUSC, and 57 (9%) were never smokers. CHAI-positive patients had significantly worse OS than CHAI-negative patients (3-year OS: 53% vs. 69%; log-rank *p* < 0.001). The biomarker remained significantly associated with OS (HR 1.70, 95% CI 1.29–2.24, *p* < 0.001) and PFI (HR 1.39, 95% CI 1.05–1.84, *p* = 0.023) in multivariable analysis. The effect was consistent across both histologies, and a model combining the CHAI score with clinicopathologic variables was well-calibrated in validation. The biomarker was not associated with stage, age, sex, or histologic subtype, and its adjusted prognostic effect was unchanged after relevant genomic alterations were added to the model. CHAI-positive tumors were, however, enriched for *KRAS* mutations (21% vs. 11% wild-type, *p* adjusted = 0.005). **Conclusions**: An H&E-only CHAI NSCLC biomarker was developed and provided independent prognostic stratification in a held-out NSCLC validation cohort. Such a biomarker has the potential to refine risk stratification in NSCLC.

## Simple Summary

Risk stratification to predict which non-small cell lung cancer (NSCLC) patients are likely to have poor outcomes remains challenging. We applied the previously developed Computational Histology Artificial Intelligence (CHAI) platform to identify a novel histologic biomarker in data from The Cancer Genome Atlas. In a validation cohort, the CHAI biomarker was associated with worse overall survival, even after clinical covariates and relevant genetic alterations in NSCLC were controlled for. The association with worse outcomes was preserved when the focus was Stage I-III disease. The biomarker was not associated with any clinical covariates, although there were a greater proportion of KRAS alterations among CHAI-biomarker-positive patients. Collectively, the CHAI NSCLC biomarker represents a novel prognostic AI-derived histologic biomarker that may improve risk stratification in NSCLC and is worthy of further study.

## 1. Introduction

Non–small cell lung cancer (NSCLC) remains a leading cause of cancer-related death worldwide despite substantial advances in targeted therapy and immunotherapy. In a recent population-based lung adenocarcinoma cohort, 3-year overall survival (OS) declined steeply with stage, from approximately 84% for Stage I to 21% for Stage IV disease [1], but outcomes within a given cancer stage are heterogeneous. Tools that further refine prognosis remain of value to patients and providers in shaping expectations and guiding management.

Biomarkers are central to NSCLC care. Evaluation of a defined set of actionable genomic alterations is recommended in the work-up of newly diagnosed advanced NSCLC to guide therapy, and these markers are increasingly relevant in earlier-stage disease. For example, adjuvant osimertinib improves outcomes in resected *EGFR*-mutant NSCLC [2,3], adjuvant alectinib in resected *ALK*-positive disease [4], and adjuvant selpercatinib in RET-positive NSCLC [5]. Beyond serving as predictive markers, several alterations can modulate expected outcomes, including susceptibility to immunotherapy [6,7]. These molecular markers complement traditional pathologic features such as grade, differentiation, and histology. Nonetheless, the identification of independent prognostic biomarkers using orthogonal methods remains an opportunity to further refine risk profiles.

Artificial intelligence applied to digitized histopathology is an emerging biomarker modality that extracts prognostic signals directly from routine H&E slides. In lung cancer, computational pathology has been used to predict actionable mutation status, immunotherapy response, and survival from whole-slide images (WSI) [8,9,10].

The Computational Histology Artificial Intelligence (CHAI) platform is a tumor-agnostic foundational system built on deep-learning tissue and cell segmentation models that quantify over 30,000 histomorphological features, such as nuclear size and morphology, cell spatial organization, immune infiltration, and stromal density, which can be combined into biomarker histologic signatures. CHAI has been applied to develop prognostic and treatment-predictive biomarkers in several other cancers including bladder [11,12,13], pancreatic [14], prostate [15], and colorectal cancers [16]; we investigated whether CHAI could develop and validate a novel prognostic biomarker, independent of established clinicopathologic factors, using data from The Cancer Genome Atlas (TCGA) lung cohorts spanning adenocarcinoma and squamous cell carcinoma.

## 2. Materials and Methods

### 2.1. Study Cohort

This study used clinical, pathologic, and digitized WSI data from two TCGA lung cancer projects: lung adenocarcinoma (TCGA-LUAD) and lung squamous cell carcinoma (TCGA-LUSC), multi-institutional efforts that collected primary NSCLC specimens with corresponding clinical and molecular data [17,18]. The study was designed and reported in accordance with REMARK guidelines [19], and the inclusion criteria stipulated at least one diagnostic H&E WSI that passed quality control on the CHAI platform.

A total of 914 patients met all criteria, comprising 450 lung adenocarcinoma (49%) and 464 lung squamous cell carcinoma (51%) cases with the date of pathologic diagnosis ranging from 1992 to 2013. The cohort encompassed the full stage distribution of NSCLC, including Stage I, II, III, and IV disease per the AJCC edition current at the time of diagnosis (ranging from the 3rd to 7th edition). Tumor grade was not available in the dataset.

**Figure 1:**
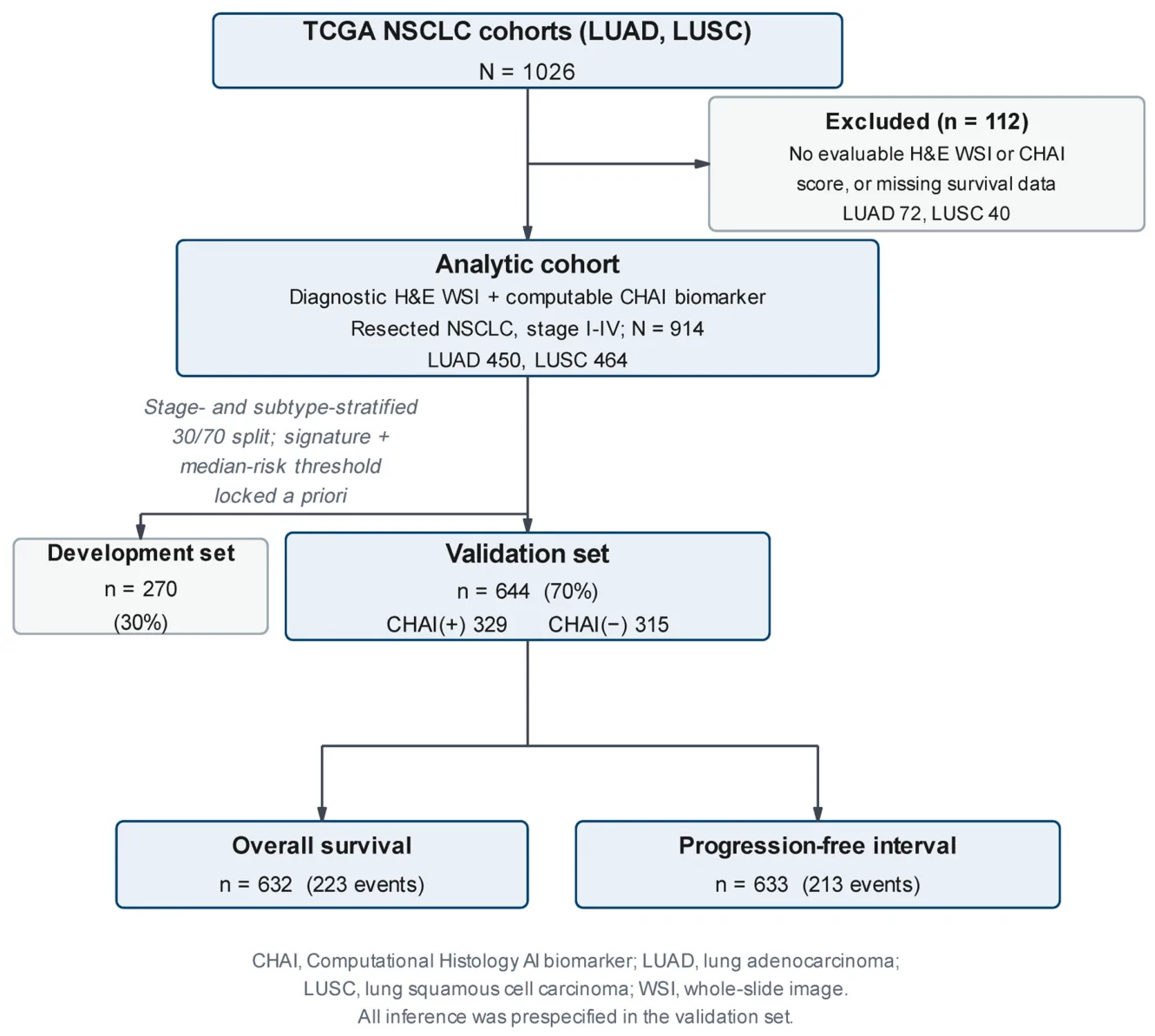
Cohort assembly and analysis. The pooled TCGA NSCLC cohort (N = 1026; TCGA-LUAD 522 and TCGA-LUSC 504) was screened; 112 patients were excluded due to the absence of evaluable diagnostic H&E WSI or failed CHAI quality control, yielding an analytic resected NSCLC cohort of N = 914 (LUAD 450; LUSC 464). Patients were stratified by stage and subtype and randomly split 30–70% into a development set (*n* = 270, 30%), in which the histologic signature and thresholds were locked, and a held-out validation set (*n* = 644, 70%; CHAI[+], 329; CHAI[−], 315), in which all reported inference was performed. A total of 632 patients had available overall survival data reported, while 633 had progression-free interval reported. CHAI, Computational Histology Artificial Intelligence Biomarker; H&E, hematoxylin and eosin; LUAD, lung adenocarcinoma; LUSC, lung squamous cell carcinoma; QC, quality control; TCGA, The Cancer Genome Atlas; WSI, whole-slide image.

To allow for independent validation in a cohort with 80% power to detect an HR of 1.5 with approximately 200 events at an event rate of 35% and to facilitate training the signature on a cohort comparable in size to that of previously locked CHAI signatures [14,15], patients were partitioned into a development set (30%, N = 270) and an independent validation set (70%, N = 644) by random sampling stratified by stage and histologic subtype (LUAD/LUSC) (Figure 1). All feature selection, model fitting, and threshold determination were performed exclusively within the development set. The resulting signature and dichotomization threshold were then locked and applied without modification to the validation set. All primary inference is reported from the validation set.

Using the methods of Hsieh and Lavori [20], we characterized the biomarker effect detectable in the validation cohort in the primary analysis for the two-sided hypothesis that the log hazard ratio (B) of the CHAI biomarker differs from 0 in a Cox proportional-hazards model of OS, where B is the coefficient for the dichotomized CHAI biomarker (H0: B = 0 versus H1: B ≠ 0). The biomarker was applied as a binary classifier with a presumed prevalence of the biomarker of 50%. With significance defined a priori at the two-sided *p* < 0.05 level and an overall OS event rate of 35% (223 events among 644 subjects), the validation cohort of *n* = 644 subjects provided 80% power to detect an interaction hazard ratio of 1.46 and 90% power to detect an interaction hazard ratio of 1.54. All subgroup analyses are reported as exploratory and hypothesis-generating.

### 2.2. CHAI Biomarker

The previously described CHAI platform [11,12,13,14,15] was applied to the TCGA lung WSIs in this study. Briefly, deep-learning tissue and cell segmentation models processed each H&E slide to extract approximately 30,000 quantitative histomorphologic features, such as nuclear size and morphology, cell spatial organization, host immune infiltration, and stromal density. The deep-learning tissue and cell segmentation components of the CHAI platform implemented in this study were previously developed through training on approximately 25,000 pan-cancer H&E-stained slides, including 500,000 nuclei annotations and 100 million µm^2^ of tissue annotated by board-certified pathologists, to segment cell and tissue types relevant to cancer biology and to quantify histomorphologic features [12]. Histomorphologic features significantly correlated with overall survival were selected using Cox proportional hazards modeling and weighted to construct one subtype-aware signature. The ultimate signature included features capturing characteristics related to immune infiltration and glandular architecture. The signature assigns each patient a continuous biomarker score. The score was dichotomized into CHAI-positive (CHAI[+], higher risk) and CHAI-negative (CHAI[−], lower risk) groups. The threshold used for dichotomization of the biomarker was determined by maximizing the hazard ratio for the biomarker in a univariable cox proportional hazards model of overall survival. Representative images of CHAI (+) and CHAI (−) status are shown in Supplementary Figure S1. The signature and threshold were locked prior to application in the held-out validation set.

### 2.3. Study Endpoints

All endpoints were measured from the date of initial pathologic diagnosis. The primary endpoint was overall survival (OS, time to death from any cause); the secondary endpoint was progression-free interval (PFI, time to disease progression, including locoregional recurrence, distant metastasis, or new primary tumor, or death with tumor, but not including death with no evidence of disease; PFI was used based on prior work validating its use as an endpoint in TCGA [21]). Patients without an event were censored at last known follow-up. Survival times were truncated at 5 years for analysis.

### 2.4. Statistical Analysis

Baseline characteristics were summarized as medians (interquartile range, IQR) for continuous variables and counts (percentages) for categorical variables and compared between CHAI groups using the Wilcoxon rank-sum test and Fisher exact test, respectively. The association of the CHAI biomarker with each endpoint was assessed by the Kaplan–Meier method and Cox proportional-hazards regression. Univariable models estimated the unadjusted association of the biomarker with each endpoint. The primary multivariable model adjusted for age, sex, AJCC stage (based on the edition in use at the time of diagnosis), smoking status, and histologic subtype (adenocarcinoma vs. squamous cell carcinoma). To evaluate whether the biomarker’s prognostic value was independent of actionable oncogenic alterations, a multivariable Cox model additionally included *EGFR*, *KRAS*, *BRAF*, and *ERBB2* mutations; *ALK*, *ROS1*, and *RET* fusions were too infrequent for stable estimation and were omitted from this model (but are reported in the association analysis below). All models were fit on complete cases given an assumption of missingness at random and limited missingness. Hazard ratios (HRs), 95% confidence intervals (CIs), and *p*-values are reported, with two-sided *p* < 0.05 considered significant. Mutation and fusion calls were obtained from the TCGA PanCancer Atlas via cBioPortal [22,23]. To evaluate calibration, a Cox model combining the CHAI score with age, sex, AJCC stage, smoking status, and histologic subtype was fit on the development set and used to predict 3-year survival in the validation set; predicted probabilities were compared with Kaplan–Meier estimates across quintiles of predicted risk.

The consistency of the biomarker’s effect was examined across subgroups (Stage I–II vs. III–IV, histologic subtype, age <65 vs. ≥65 years, and smoking status) by fitting the univariable Cox model within each and testing CHAI×subgroup interaction terms.

Whether CHAI positivity tracked any single known feature was examined by comparing CHAI-positive prevalence across clinicopathologic and genomic subgroups (Wilson 95% confidence intervals), with mutant-versus-wild-type comparisons tested by the Fisher exact test and clinicopathologic balance. A broader set of 13 recurrent genomic alterations in lung cancer was included, beyond the set of actionable mutations with higher frequency included in the multivariable survival analysis above; the Benjamini–Hochberg method was used to adjust for multiple hypothesis testing. Time-dependent discrimination of the continuous CHAI percentile score was assessed by the cumulative/dynamic time-dependent area under the ROC curve with inverse-probability-of-censoring weighting [24].

Analyses were performed in R version 4.4.3.

## 3. Results

### 3.1. Patient Characteristics

A total of 914 patients met the inclusion criteria; a stage and histologic subtype-balancing split was done, and 270 (30%) patients were assigned to the development set and 644 (70%) to the held-out validation set (Supplementary Table S1). In the validation cohort, the median age was 68 years (IQR 60–74), 388 patients (60%) were male, and the cohort was balanced between subtypes (LUAD 317 [49%], LUSC 327 [51%]). The majority of patients were former smokers (417, 65%); 160 (25%) were active smokers, and 57 (9%) were never smokers. Disease was distributed across AJCC stages (I, 334 [52%]; II, 182 [28%]; III, 97 [15%]; IV, 23 [4%]). The CHAI biomarker classified 329 patients (51%) as higher risk CHAI[+] and 315 (49%) as lower risk CHAI[−]. Over follow-up, the validation set accrued 223 deaths (OS; median follow-up 23.1 months among censored patients) and 213 progression events (PFI). Baseline characteristics were well balanced between CHAI groups except for age (*p* = 0.047) and are shown in Table 1.

### 3.2. CHAI Biomarker and Survival

**Figure 2:**
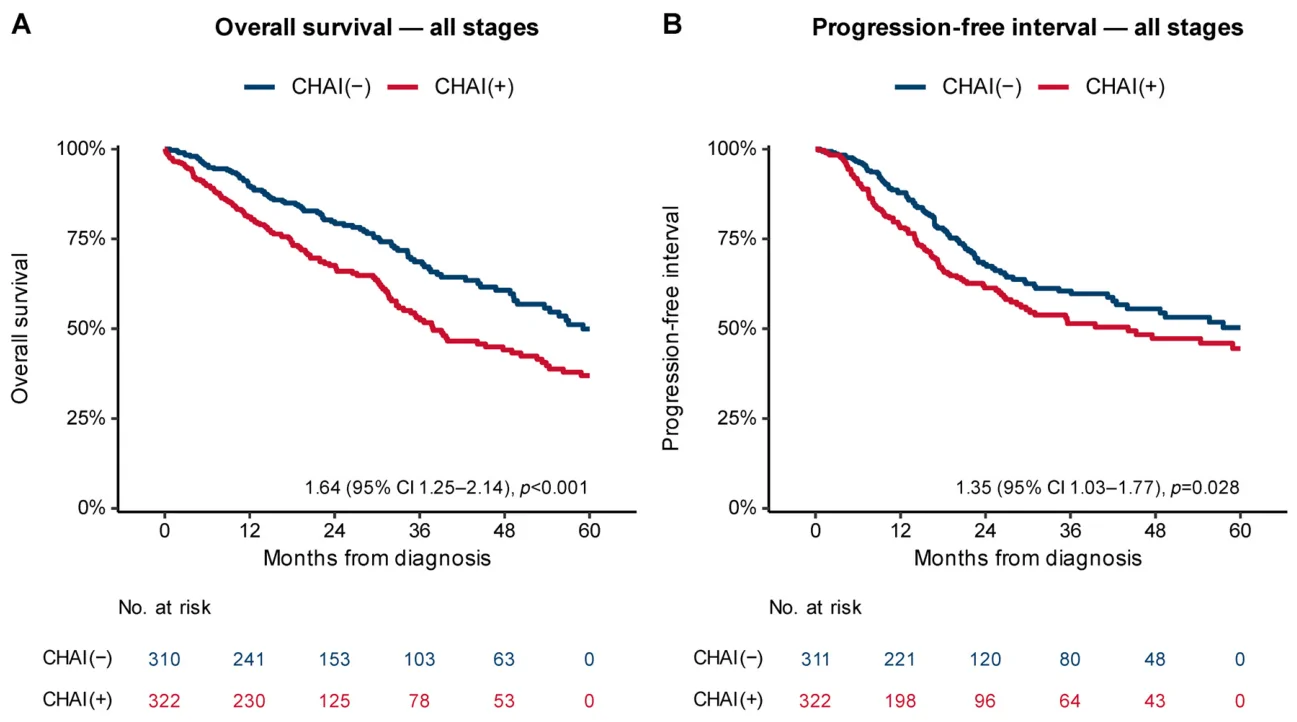
Kaplan–Meier survival in the validation cohort by CHAI group. (**A**) Overall survival (primary endpoint). A total of 632 participants had available overall survival data for analysis. (**B**) Progression-free interval (secondary endpoint). A total of 633 participants had available progression-free interval data for analysis. Univariable hazard ratio (95% CI), Cox *p*-values are labeled.

**Figure 3:**
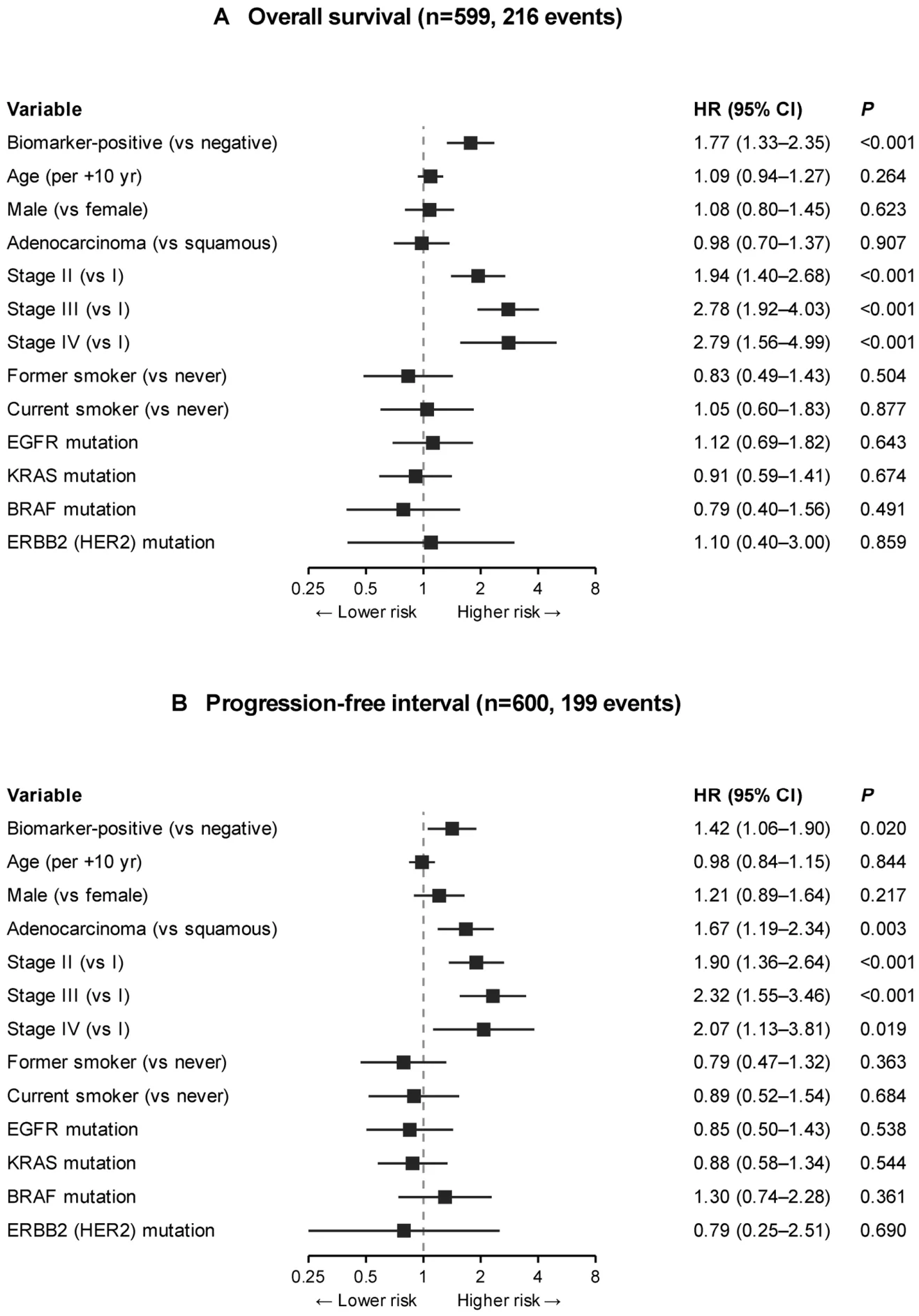
Multivariable Cox proportional-hazards models including genomic variants. Adjusted hazard ratios for (**A**) overall survival and (**B**) progression-free interval in the validation cohort from Cox models including the CHAI biomarker, age, sex, smoking status, AJCC stage, histologic subtype, and recurrent genomic alterations (EGFR, KRAS, BRAF, ERBB2 mutations). *ALK*, *ROS1*, and *RET* fusions were too infrequent for stable estimation.

CHAI-positive patients had significantly worse outcomes for both endpoints (3-year OS 53.1% vs. 68.6%, log-rank *p* < 0.001, Figure 2A; 3-year PFI 51.4% vs. 60.5%, *p* = 0.028, Figure 2B). According to univariable Cox regression, the biomarker was associated with increased hazards of OS (HR 1.64, 95% CI 1.25–2.14, *p* < 0.001) and PFI (HR 1.35, 95% CI 1.03–1.77, *p* = 0.028; Table 2), and it remained independent after adjustment for age, sex, smoking status, AJCC stage, and histologic subtype (OS HR 1.70, 95% CI 1.29–2.24, *p* < 0.001, *n* = 612 available for complete case analysis; PFI HR 1.39, 95% CI 1.05–1.84, *p* = 0.023, *n* = 613 available for complete case analysis; Table 2). Adding actionable genomic alterations into the multivariable survival model left these estimates essentially unchanged (OS HR 1.77, 95% CI 1.33–2.35, *p* < 0.001, *n* = 599 available for complete case analysis; PFI HR 1.42, 95% CI 1.06–1.90, *p* = 0.020, *n*= 600 available for complete case analysis; Figure 3), with AJCC stage being the only other major determinant of outcome, adenocarcinoma histology being a higher risk for PFI but not OS, and no individual alteration being independently prognostic. Discrimination was modest but stable across follow-up, with time-dependent AUCs of approximately 0.59–0.62 for OS and 0.54–0.64 for PFI between 6 and 54 months. A sensitivity analysis of the multivariable model focusing on localized disease (excluding Stage IV) produced largely similar results (Supplementary Figure S2).

**Figure 4:**
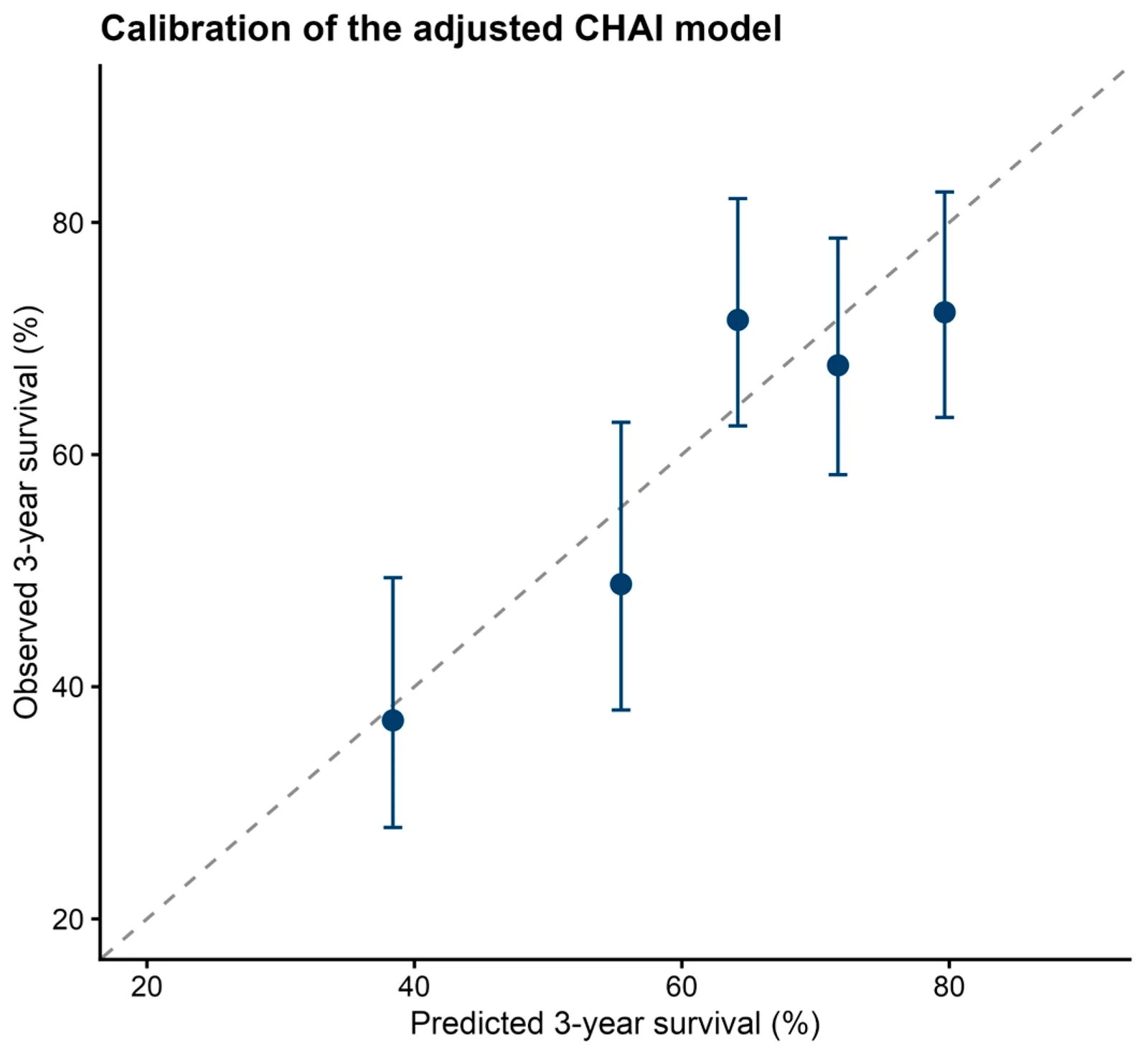
Calibration of the adjusted CHAI prognostic model (validation cohort). A Cox model combining the CHAI percentile score with age, sex, smoking status, AJCC stage, and histologic subtype was fitted on the development set and used, without refitting, to predict 3-year survival for each validation patient. Points show mean model-predicted versus Kaplan–Meier-observed 3-year survival across quintiles of predicted risk (error bars, 95% CI for observed); the dashed line indicates perfect calibration.

To test whether the prognostic biomarker is quantitatively informative, a prognostic model combining the continuous score with other baseline clinical variables (age, sex, smoking status, AJCC stage, and histologic subtype) was fit on the development set and used to predict each validation patient’s 3-year survival; without any refitting, predicted probabilities agreed closely with Kaplan–Meier-observed survival across quintiles of predicted risk (validation C-index 0.63; Figure 4). The slope and intercept of the calibration plot for 3-year OS were 0.78 (95% CI: 0.45 to 1.12) and −0.06 (95% CI: −0.34 to 0.21), respectively.

### 3.3. Performance Across Clinical Subgroups

Due to the TCGA cohort’s clinical heterogeneity, the biomarker’s prognostic effect was assessed across clinically important subgroups (Supplementary Figure S3). The CHAI biomarker was significantly associated with OS within both early-stage (I–II) (HR 1.56, 95% CI 1.14–2.15) and later-stage (III–IV) disease (HR 2.07, 95% CI 1.25–3.44) in both histologies (LUAD HR 1.58, 95% CI 1.07–2.35; LUSC HR 1.69, 95% CI 1.17–2.43) and in both younger and older patients (<65 y HR 1.89, 95% CI 1.20–2.98; ≥65 y HR 1.49, 95% CI 1.07–2.08). The association of the CHAI biomarker with OS was maintained among former smokers (the largest smoking subgroup), although it was not significant in the never-smoker or active-smoker groups with fewer events. Tests for interaction were nonsignificant for stage, subtype, smoking status, and age (all *p* > 0.1).

### 3.4. CHAI Association with Clinical and Molecular Characteristics

**Figure 5:**
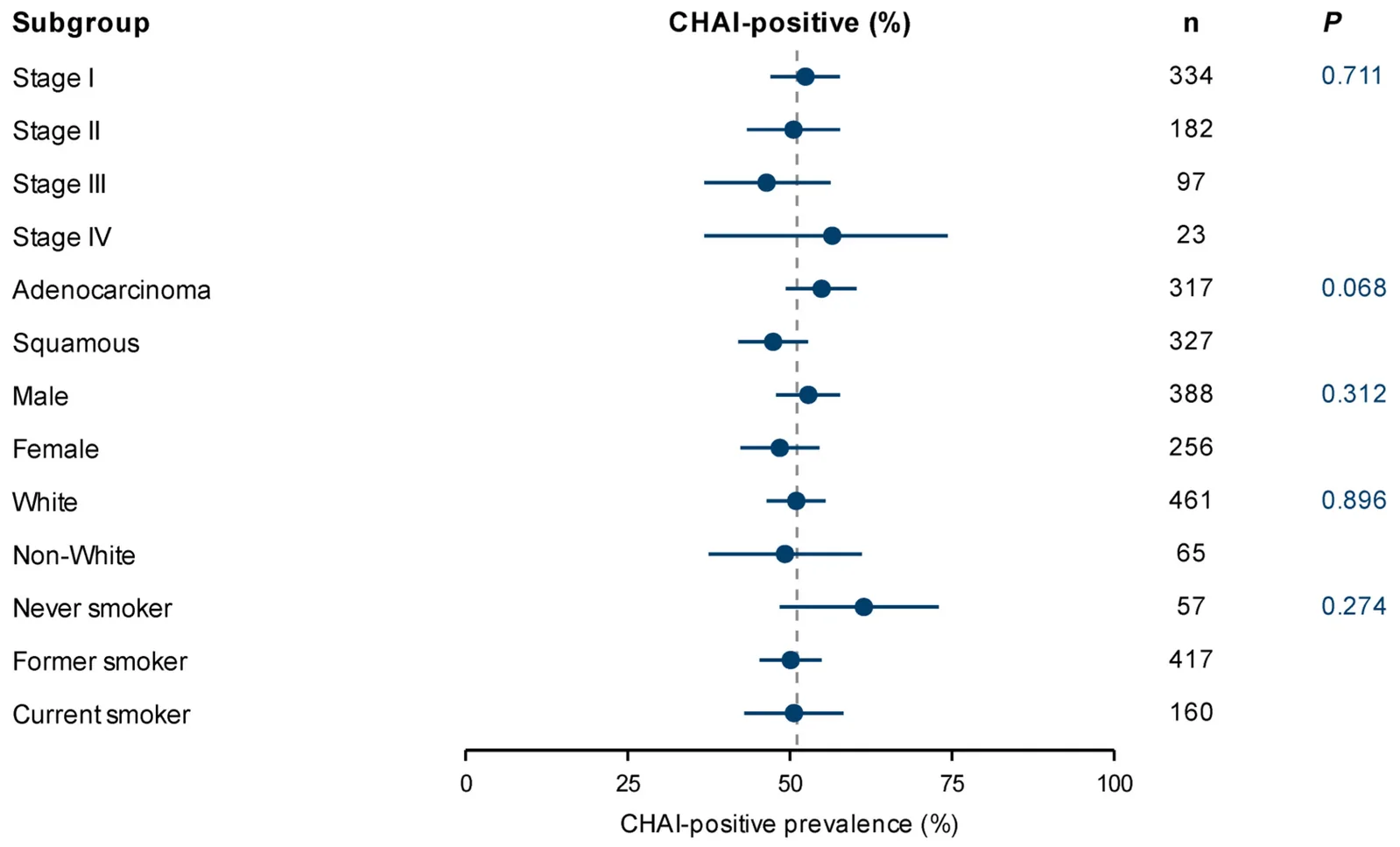
CHAI-positive prevalence across clinicopathologic subgroups (validation cohort). Each point is the percentage of CHAI biomarker-positive patients within a subgroup, with a 95% confidence interval; the dashed line marks the overall cohort prevalence (51%). Prevalence is statistically indistinguishable from the cohort rate across every clinicopathologic stratum. *p* values correspond to chi-squared (for multi-level variable)/Fisher’s exact (for binary variable) tests of association between each factor and CHAI status. *ALK*, *ROS1*, and *RET* fusions (*n* ≤ 4 each) were too rare to estimate.

To evaluate whether CHAI positivity simply tracks any single known feature, we evaluated CHAI-positive prevalence rate to the cohort rate (51%) by subgroup. It did not differ significantly across AJCC stage (χ^2^ *p* = 0.7), histologic subtype (*p* = 0.07), sex (*p* = 0.3), race (*p* > 0.9), or smoking status (*p* = 0.27) (Figure 5).

When assessing for genomic alterations associated with CHAI positivity, KRAS alterations were found to be associated with CHAI-biomarker-positive status (KRAS: CHAI + 21% vs. CHAI − 11%), while p53 alterations were more common among CHAI-negative tumors (CHAI + 62% vs. CHAI − 75%) (Supplementary Figure S4). In contrast, no association with the CHAI biomarker was noted for STK11, KEAP1, PIK3CA, EGFR, BRAF, MET, ERBB2, NRAS, ROS1, ALK, or RET.

## 4. Discussion

It was shown a decade ago that histomorphologic features extracted from whole-slide images can stratify overall survival in resected NSCLC independently of stage and grade [10]; more recently, weakly supervised models inferred tumor-microenvironment composition from H&E WSIs and predicted immune-checkpoint-inhibitor benefit in NSCLC [8]. These prior works show that routine histology encodes prognostic and predictive signals beyond stage and molecular annotation, motivating the exploration of a novel histology-only modality for biomarker discovery.

In a TCGA LUAD and LUSC mixed-stage validation cohort, a CHAI biomarker derived solely from the diagnostic H&E slide independently stratified the risk for both shorter overall survival and progression-free interval after adjustments were made for age, sex, AJCC stage, smoking status, and histologic subtype. The biomarker was not associated with stage, age, sex, smoking status, or histologic subtype, supporting an orthogonal signal. It should be noted that AJCC stage in this dataset was labeled with variable editions of the AJCC, complicating assessments of independence from stage. Future studies will evaluate the biomarker in early and late stage disease. Interestingly, there was an association observed with KRAS alterations, although the relationship between CHAI biomarker status and OS was independent of KRAS alteration status in multivariable modeling. Previous work has suggested that specific morphologic patterns, such as mucinous histology, poor differentiation status, and inflammatory infiltrate, may be more common among KRAS-mutated NSCLC, although this in turn can be influenced by co-mutations [25,26]. However, future study in larger datasets are needed to isolate the mechanistic links between the KRAS alterations and the CHAI biomarker. Surprisingly, p53 alteration was associated with CHAI (−) status; while counterintuitive, potential explanations include heterogeneity in mutation types among p53 alterations as well as variable co-occurrence with driver mutations. Additional study will be needed in future cohorts to better define and understand this association. Because the two major histologies differ considerably in morphology and biology, the CHAI signature was developed subtype-aware, and its prognostic effect was consistent across both histologies, stage groups, and age groups. A model combining the score with standard clinicopathologic variables, locked on the development set, was well calibrated in the independent validation set.

These findings address a recognized gap. Anatomic stage remains the dominant prognostic determinant in NSCLC, yet outcomes within a stage are heterogeneous, and implementation of novel prognostic molecular tools require additional tissue, cost, and assay turnaround. Refining post-operative risk, for example, has become more consequential with increased use of adjuvant targeted therapy (osimertinib for *EGFR*-mutant, alectinib for *ALK*-positive disease, and selpercatinib in RET-positive disease) and adjuvant immunotherapy, intensifying the need to identify patients most likely to recur [3,4,5,27,28]. It is important to note, however, that the CHAI biomarker was not designed in this study to be treatment-predictive, and data was not available to test any treatment-predictive effects of the biomarker. Nevertheless, it may improve understanding of prognosis to shape personalization of decisions and expectations for patients in the evolving localized NSCLC landscape. Unlike molecular or gene-expression classifiers, the CHAI biomarker requires no additional tissue and is computed from material already generated from every resection. In addition, it requires no additional staining (utilizing routinely collected H&E-stained slides) and may have the potential for superior turnaround times with lower cost than traditional molecular tests [29,30]. These characteristics make it an appealing possible tool, and it may complement existing efforts with alternative biomarker modalities, such as circulating tumor DNA [31,32]. External validation of the CHAI biomarker in contemporary cohorts with modern treatment paradigms will be an important further step to further test the biomarker’s validity and clinical utility.

A number of limitations temper interpretation. This is a retrospective analysis of a single data source (TCGA) which carries a risk of optimism bias. Additionally, participants in the study cohort were diagnosed and treated across a range of time from 1992 to 2013, with considerable evolution in treatment paradigms, staging, and diagnostics occurring since then; future validation efforts should evaluate the model on more contemporary cohorts. The cohort was managed predominantly by surgery in an era preceding modern adjuvant targeted therapy and immunotherapy, and systemic treatment was not reliably annotated, so the biomarker was evaluated as a prognostic marker rather than a predictor of benefit from any specific therapy; its predictive utility for specific adjuvant therapies is an untested and important next question. Additionally, the cohort included patients diagnosed prior to the standard use of adjuvant chemotherapy and universally preceded the advent of modern neoadjuvant and perioperative systemic therapy approaches incorporating immunotherapy [33]; whether this biomarker might be effective with resected specimens after neoadjuvant treatment remains to be seen. There was limited annotation of traditional histologic characteristics such as grade in this dataset; future studies would benefit from benchmarking the CHAI biomarker against such traditional histopathologic characteristics. Comparisons of the biomarker to other potential prognostic tests such as ctDNA, PD-L1 status, and tumor mutational burden were not possible in this study given the data available in the TCGA but may be of interest in future studies. The time-dependent AUC of the CHAI biomarker suggested modest discriminatory performance for OS; further study will be needed to assess the clinical utility of the biomarker in real-world practice. This study was not powered to support sub-group analyses, and so all subgroup analyses including those of smoking status and p53 and KRAS alterations must be viewed as primarily hypothesis generating with further evaluation needed to clarify associations with subgroups. Relatedly, the study was not of adequate size with sufficient frequency of genomic alterations in ALK, ROS1, and RET to allow for incorporation of these alterations in the multivariable model, and future study will need to investigate whether the CHAI biomarker is associated with and independent from these genomic alterations. PFI, while well-documented for its use in TCGA studies, is an imperfect outcome for recurrence, meaning that further study will be needed to test associations between the CHAI biomarker and recurrence-free survival. The TCGA dataset is limited in granular information available regarding WSI preparation (for example scanner use), limiting assessments for batch effects related to scanning or specimen preparation. Finally, staging annotations are complicated by the challenge of variable AJCC editions used across the specimen collection interval, making robust stage sub-group and tests of independence from stage challenging.

## 5. Conclusions

In summary, a histology-only CHAI biomarker provided independent prognostic stratification across NSCLC subtypes, stages, and endpoints in a locked validation cohort. If confirmed externally, such a biomarker could refine post-operative risk assessment and help target emerging adjuvant therapy to the patients most likely to benefit.

## Figures and Tables

**Table 1:** Baseline characteristics of the validation cohort by CHAI group. Demographic and clinicopathologic characteristics of the held-out validation cohort (N = 644), shown overall and stratified by the locked CHAI biomarker call (CHAI[−], lower risk, *n* = 315; CHAI[+], higher risk, *n* = 329). Continuous variables are summarized as median (interquartile range) and categorical variables as *n* (%). Wilcoxon rank-sum test was used for continuous variables, while the Fisher exact test was used for categorical variables, with *p*-values being reported. AJCC, American Joint Committee on Cancer (assigned based on the edition current at the time of pathologic diagnosis); CHAI, Computational Histology Artificial Intelligence Biomarker; IQR, interquartile range; LUAD, lung adenocarcinoma; LUSC, lung squamous cell carcinoma.

Characteristic	CHAI(−) (N = 315)	CHAI(+) (N = 329)	*p*
Age, median (IQR), years	67 (60–73)	69 (60–74)	0.047
Sex			0.295
Female	132 (42%)	124 (38%)	
Male	183 (58%)	205 (62%)	
Race			0.887
White	226 (72%)	235 (71%)	
Black or African American	28 (9%)	25 (8%)	
Asian	5 (2%)	7 (2%)	
American Indian or Alaska Native	0 (0%)	0 (0%)	
Not reported	56 (18%)	62 (19%)	
Smoking status			0.379
Never	22 (7%)	35 (11%)	
Former	208 (66%)	209 (64%)	
Current	79 (25%)	81 (25%)	
Not reported	6 (2%)	4 (1%)	
Histologic subtype			0.059
LUAD	143 (45%)	174 (53%)	
LUSC	172 (55%)	155 (47%)	
AJCC pathologic stage			0.847
Stage I	159 (50%)	175 (53%)	
Stage II	90 (29%)	92 (28%)	
Stage III	52 (17%)	45 (14%)	
Stage IV	10 (3%)	13 (4%)	
Not reported	4 (1%)	4 (1%)	

**Table 2:** Univariable and multivariable Cox proportional-hazards results for the CHAI biomarker. Hazard ratios are reported for CHAI(+) versus CHAI(−) for overall survival and progression-free interval. Multivariable models adjust for age, sex, smoking status, AJCC pathologic stage (I–IV), and histologic subtype (LUAD vs. LUSC). Univariable models included all patients with the relevant endpoint (overall survival, *n* = 632; progression-free interval, *n* = 633); multivariable models additionally excluded patients with missing age or unreported stage (*n* = 612 and *n* = 613, respectively). CI, confidence interval; HR, hazard ratio; OS, overall survival; PFI, progression-free interval.

Endpoint	Univariable HR (95% CI)	Multivariable HR (95% CI)
OS	1.64 (1.25–2.14), *p* < 0.001	1.70 (1.29–2.24), *p* < 0.001
PFI	1.35 (1.03–1.77), *p* = 0.029	1.39 (1.05–1.84), *p* = 0.023

## Data Availability

All histologic data from the TCGA used for analysis are publicly available from the TCGA. Biomarker calls and code used for statistical analyses may be made available to qualified researchers upon reasonable request to the corresponding author.

## Supplementary Materials

The following supporting information can be downloaded at https://www.mdpi.com/article/10.3390/cancers18182916/s1: Figure S1. Representative images of CHAI biomarker status. Representative images of adenocarcinoma CHAI (+) and CHAI (−) as well as squamous cell carcinoma CHAI (+) and CHAI (−) samples are presented; Figure S2: Multivariable Cox proportional-hazards models excluding Stage IV; Figure S3: CHAI biomarker and overall survival by subgroup (validation cohort); Figure S4: CHAI associations with genomic alterations (validation cohort); Table S1: Characteristics across the development and validation cohorts.

## Abbreviations

The following abbreviations are used in this manuscript:

NSCLC	Non-small cell lung cancer
CHAI	Computational histology artificial intelligence
H&E	Hematoxylin and eosin
TCGA	The Cancer Genome Atlas
LUAD	Lung adenocarcinoma
LUSC	Lung squamous cell carcinoma
WSI	Whole-slide image
OS	Overall survival
PFI	Progression-free interval
HR	Hazard ratio
IQR	Interquartile range
