# Supplementary material for: A Computational Histology Artificial Intelligence Prognostic Biomarker in Non-Small Cell Lung Cancer Using the Cancer Genome Atlas
Source: Cancers (Basel). 2026 Sep 9;18(18):2916. doi: 10.3390/cancers18182916 (PMC13604284; doi:10.3390/cancers18182916)
Supplement: Supplementary file 1 [file cancers-18-02916-s001.zip › cancers-4534295-supplementary.pdf]

cancers

MDPI

Supplementary Materials: A Computational Histology Artificial Intelligence Prognostic Biomarker in Non–Small Cell Lung Cancer Using The Cancer Genome Atlas

Benjamin A Bleiberg , Masaoki Ito , Melina Marmarelis , Viswesh Krishna , Vivek Nimgaonkar , Haochen Zhang , Trevor J. Royce , Yasuhiro Tsutani , David Kozono , Charu Aggarwal

Table S1. Characteristics across the development and validation cohorts. Demographic and clinicopathologic characteristics are reported for the development (n=270) and validation cohorts (n=644). Continuous variables are summarized as median (interquartile range) and categorical variables as n (%). Wilcoxon rank-sum test was used for continuous variables; Fisher exact test for categorical variables, reporting p-values. AJCC, American Joint Committee on Cancer (AJCC stage was recorded at the time of pathologic diagnosis); CHAI, Computational Histology artificial-intelligence biomarker; IQR, interquartile range; LUAD, lung adenocarcinoma; LUSC, lung squamous cell carcinoma.

Characteristic	Development (N=270)	Validation (N=644)	P
**Age, median (IQR), years**	66 (60-73)	68 (60-74)	0.158
**Sex**			0.866
Female	105 (39%)	256 (40%)	
Male	165 (61%)	388 (60%)	
**Race**			0.622
White	196 (73%)	461 (72%)	
Black or African American	24 (9%)	53 (8%)	
Asian	5 (2%)	12 (2%)	
American Indian or Alaska Native	1 (0%)	0 (0%)	
Not reported	44 (16%)	118 (18%)	
**Smoking Status**			0.917
Never	23 (9%)	57 (9%)	
Former	170 (63%)	417 (65%)	
Current	73 (27%)	160 (25%)	
Not reported	4 (1%)	10 (2%)	
**Histologic subtype**			1.000
LUAD	133 (49%)	317 (49%)	
LUSC	137 (51%)	327 (51%)	
**AJCC pathologic stage**			0.997
Stage I	141 (52%)	334 (52%)	
Stage II	77 (29%)	182 (28%)	
Stage III	41 (15%)	97 (15%)	
Stage IV	8 (3%)	23 (4%)	
Not reported	3 (1%)	8 (1%)	

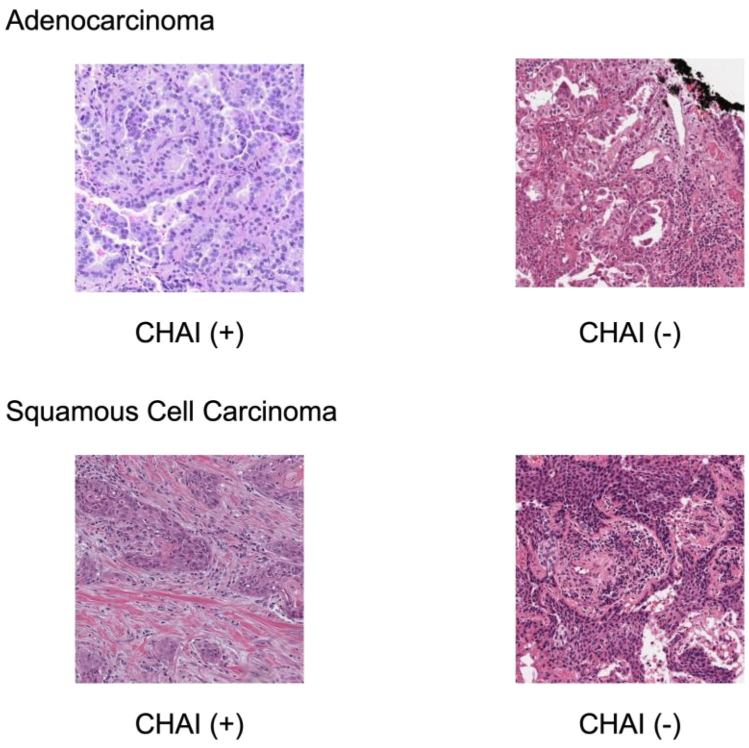

Figure S1. Representative images of CHAI biomarker status. Representative images of adenocarcinoma CHAI (+) and CHAI (-) as well as squamous cell carcinoma CHAI (+) and CHAI (-) samples are presented.

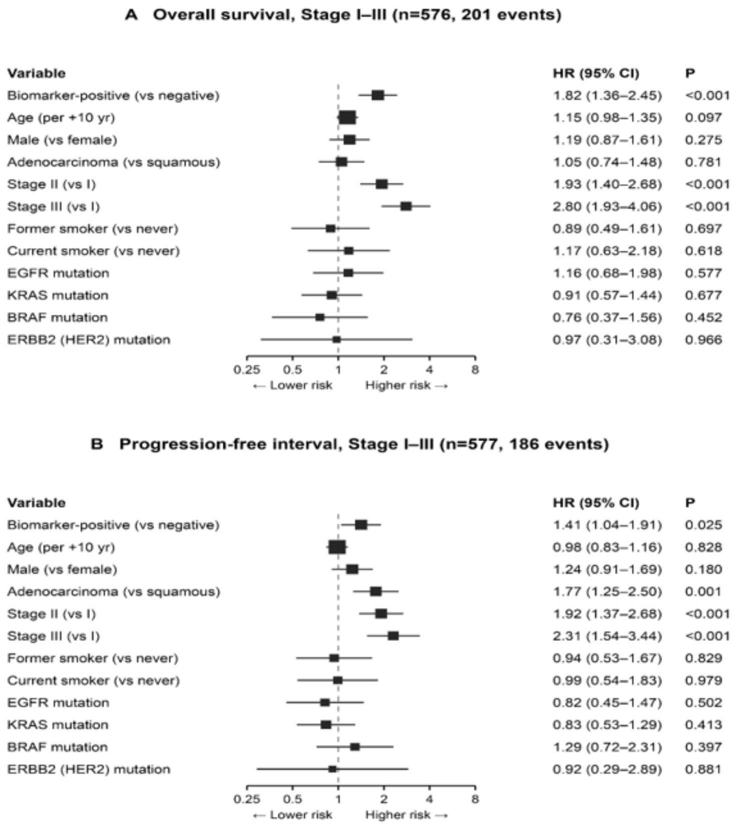

Figure S2. Multivariable Cox proportional-hazards models excluding Stage IV. Adjusted hazard ratios for (A) overall survival and (B) progression-free interval in the validation cohort, from Cox models including the CHAI biomarker, age, sex, smoking status, AJCC stage, histologic subtype,

and recurrent genomic alterations (EGFR, KRAS, BRAF, ERBB2 mutations), excluding patients with Stage IV disease. ALK, ROS1, and RET fusions were too infrequent for stable estimation and are not shown.

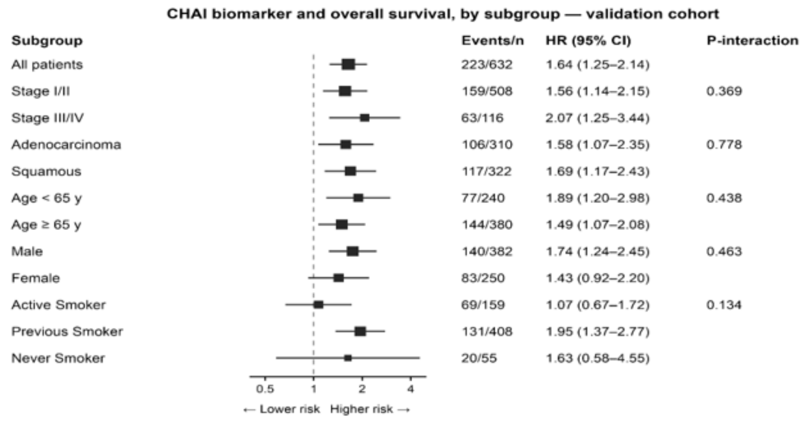

Figure S3. CHAI biomarker and overall survival by subgroup (validation cohort). Hazard ratios for overall survival associated with CHAI biomarker status, estimated within each subgroup by univariable Cox regression. Squares are hazard ratios (sized by precision) with 95% confidence intervals on a log scale. Events/n and the interaction p-values are shown.

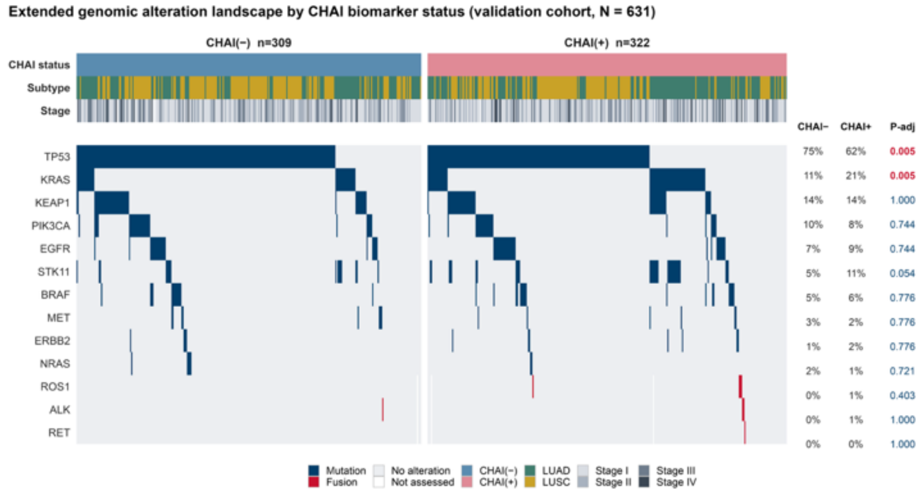

Figure S4. CHAI associations with genomic alterations (validation cohort). Oncoprint of the 13 alterations analyzed in this study (*TP53*, *KRAS*, *KEAP1*, *PIK3CA*, *EGFR*, *STK11*, *BRAF*, *MET*, *ERBB2*, and *NRAS* mutations in navy; *ALK*, *ROS1*, and *RET* fusions in red) in the 631 of 644 validation patients with genomic profiling. Each column is one patient, split into CHAI-negative (n = 309) and CHAI-positive (n = 322) blocks and ordered within block by alteration pattern; genes are ordered by frequency. Light gray, profiled with no event; white, not assessed (fusion calls available for 628 patients). Tracks above show CHAI status, histologic subtype, and AJCC stage. Right-hand columns give per-group prevalence and unadjusted Fisher exact adjusted *P* values, adjusted using the Benjamini-Hochberg method.
